# Application of multi-omics data integration and machine learning approaches to identify epigenetic and transcriptomic differences between *in vitro* and *in vivo* produced bovine embryos

**DOI:** 10.1371/journal.pone.0252096

**Published:** 2021-05-24

**Authors:** Maria B. Rabaglino, Alan O’Doherty, Jan Bojsen-Møller Secher, Patrick Lonergan, Poul Hyttel, Trudee Fair, Haja N. Kadarmideen

**Affiliations:** 1 Quantitative Genetics, Bioinformatics and Computational Biology Group, Department of Applied Mathematics and Computer Science, Technical University of Denmark, Lyngby, Denmark; 2 School of Agriculture and Food Science, University College Dublin, Dublin, Ireland; 3 Department of Veterinary and Animal Sciences, University of Copenhagen, Frederiksberg C, Denmark; University of Florida, UNITED STATES

## Abstract

Pregnancy rates for *in vitro* produced (IVP) embryos are usually lower than for embryos produced *in vivo* after ovarian superovulation (MOET). This is potentially due to alterations in their trophectoderm (TE), the outermost layer in physical contact with the maternal endometrium. The main objective was to apply a multi-omics data integration approach to identify both temporally differentially expressed and differentially methylated genes (DEG and DMG), between IVP and MOET embryos, that could impact TE function. To start, four and five published transcriptomic and epigenomic datasets, respectively, were processed for data integration. Second, DEG from day 7 to days 13 and 16 and DMG from day 7 to day 17 were determined in the TE from IVP vs. MOET embryos. Third, genes that were both DE and DM were subjected to hierarchical clustering and functional enrichment analysis. Finally, findings were validated through a machine learning approach with two additional datasets from day 15 embryos. There were 1535 DEG and 6360 DMG, with 490 overlapped genes, whose expression profiles at days 13 and 16 resulted in three main clusters. Cluster 1 (188) and Cluster 2 (191) genes were down-regulated at day 13 or day 16, respectively, while Cluster 3 genes (111) were up-regulated at both days, in IVP embryos compared to MOET embryos. The top enriched terms were the KEGG pathway "focal adhesion" in Cluster 1 (FDR = 0.003), and the cellular component: "extracellular exosome" in Cluster 2 (FDR<0.0001), also enriched in Cluster 1 (FDR = 0.04). According to the machine learning approach, genes in Cluster 1 showed a similar expression pattern between IVP and less developed (short) MOET conceptuses; and between MOET and DKK1-treated (advanced) IVP conceptuses. In conclusion, these results suggest that early conceptuses derived from IVP embryos exhibit epigenomic and transcriptomic changes that later affect its elongation and focal adhesion, impairing post-transfer survival.

## Introduction

Assisted reproductive technologies (ART) have been used in cattle breeding since the 1950s, generating millions of healthy animals since then [1–3]. After artificial insemination (AI), the ART most frequently implemented in cattle reproduction is embryo transfer, where embryos are produced either (i) *in vivo* by ovarian superstimulation leading to multiple ovulations followed by AI, embryo collection and transfer (MOET) or (ii) *in vitro* embryo production (IVP), involving maturation and fertilization of oocytes collected from live animals via transvaginal aspiration of follicles or by recovery from the ovaries after slaughter.

Amongst livestock species, ART are used to the greatest extent in cattle, due to their economic importance and the relative ease with which the reproductive tract can be manipulated. Furthermore, in terms of the commercial embryo transfer industry, embryo production has shifted from MOET to IVP during the last few years [4]. However, IVP embryos differ in several characteristics (morphological, ultrastructural, physiological, transcriptional, and metabolic) from those derived by MOET, which can impact their survival [5]. A comprehensive review of the post-transfer consequences of IVP embryos noted that such embryos yielded around ~25% lower pregnancy rates compared with MOET embryos, according to the results of 12 studies performed from 1992 to 2014 [6]. Pregnancy losses were more prevalent early in gestation; around 40% of cows receiving an IVP embryo at day 7/8 post-oestrus were no longer pregnant at day 18 to 21 of gestation. One potential factor influencing embryo survival is impaired post-hatching elongation of IVP embryos, which begins at around day 13 of pregnancy [7]. Around this time, the trophectoderm (TE) begins to secrete interferon-tau, the pregnancy recognition factor in cattle [8]. Conceptuses derived from the transfer of IVP conceptuses were smaller at day 13 than their MOET counterparts [9], but were similar in length at day 16 and 17 [10, 11]. This suggests that initiation of elongation might be impaired in IVP embryos; moreover, for embryos that succeed to elongate, this process may start slowly and eventually "catch up" with MOET embryos. Furthermore, it has been demonstrated that the transcriptomic response of the endometrium differs between MOET- and IVP-derived conceptuses both *in vivo* [12, 13] and *in vitro* [14].

Several studies have shown that the use of ART can impact on the embryo transcriptome, both at the blastocyst stage [15–19], and after elongation [20], or in the transition from a spherical to an ovoid blastocyst [21]. Alterations in the transcriptomic profile are manifested in the capacity of the blastocyst to sustain development to term, for both MOET [22] and IVP embryos [23]. The embryo depends on the expression of certain genes encoding for key proteins required to undergo sequential development, including differentiation and lineage commitment, as well as appropriate temporal communication with the female reproductive tract leading to maternal recognition of pregnancy. ART-induced transcriptomic aberrancies may hamper the availability of gene products required for further embryo development.

The divergent transcriptomic profile is likely to be also associated with altered epigenetic regulation [24], defined as heritable changes in gene activity or function without changes in the DNA sequence [25]. DNA methylation is one of the best-characterized epigenetic mechanisms primarily known to influence gene expression [26]. Usually, hypermethylation of CpG islands in promoters is related to transcriptional suppression [27]. However, several studies have shown that gene body methylation is also strongly involved in gene expression [28–30], with a bias for exonic regions [31]. Furthermore, epigenetic modifications can be induced by several external factors [32], affecting IVP and MOET embryos differentially. For example, known factors including the *in vitro* culture medium used [33], and paternal characteristics, such as bull age [34], have been shown to impact at the blastocyst stage. Differential methylation was reported to be most prominent at intragenic sequences within the TE of IVP and MOET embryos, but not in the embryonic disc in elongated-day 17-embryos compared to similar stage embryos produced by AI [35]. Accordingly, abnormalities in the TE, the outermost layer of the conceptus that is in contact with the endometrium, may lead to impaired conceptus elongation.

Undoubtedly, these studies have helped to shed light on the underlying biological mechanisms in the IVP embryo that compromise pregnancy success. However, one of the remaining unanswered questions is whether transcriptomic aberrancies are exclusively related to the *in vitro* process *per se* or are induced by the lack of exposure to the oviductal and uterine environment, regardless of variables such as the maturation media, technical procedures, or parental characteristics. In addition, apart from one [21], these studies have compared the transcriptome or epigenome of embryos *at the same stage*. Therefore, an analysis integrating different sources of transcriptomic and epigenomic data of bovine embryos before and after elongation could unravel crucial differences between IVP-derived embryos and those derived by MOET.

The objective of the present study was to apply a multi-omics data integration approach to identify genes that are temporally differentially expressed and differentially methylated in the exonic regions between IVP and MOET embryos from the blastocyst stage to the elongated conceptus, that could impact on TE function. To accomplish this aim, publicly available data were integrated and re-analysed using a range of bioinformatics tools. In addition, to underpin our findings, the main results were employed to make predictions in additional independent external data, using machine-learning methods.

## Materials and methods

**Fig 1:**
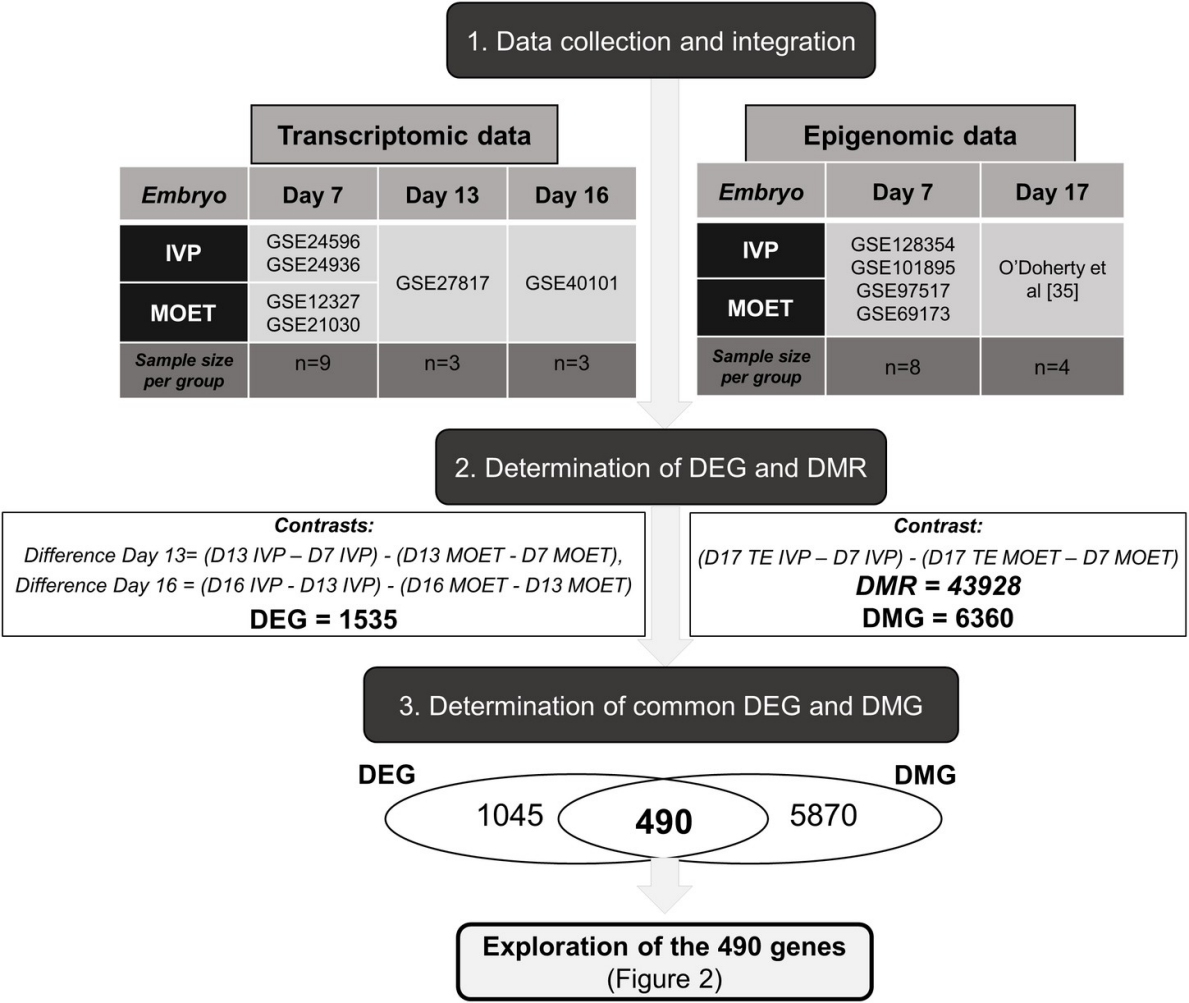
Pipeline of the methodology followed in this study. Step 1: Datasets with the accession number GSE# were downloaded from the Gene Expression Omnibus (GEO) or were obtained from a previous study [35] and processed for data integration. Step 2. Temporally differentially expressed genes (DEG) from day 7 (D7) to D13 and D16 and differentially methylated regions (DMR) in the trophectoderm (TE) from D7 to D17 were determined between groups. Differentially methylated genes (DMG) were the genes in the corresponding exons of the DMR. Step 3. DEG and DMG were overlapped to determine common genes, which were further analysed (Fig 2). Each step is explained in detail in the methodology section. IVP: embryos produced *in vitro*. MOET: embryos produced *in vivo* (after ovarian superovulation).

The pipeline followed in this study is shown in Fig 1. Each step is explained in detail below.

### Step 1: Embryo data collection and bioinformatic processing

Both transcriptomic and epigenomic datasets were downloaded from a public functional genomic data repository: Gene Expression Omnibus (GEO) from the National Center for Biotechnology Information [36, 37]. The R software platform [38] was employed for all bioinformatics procedures.

#### Transcriptomic data

Six datasets were employed to obtain transcriptomic data from MOET and IVP embryos. Data from the following types of embryo were downloaded from:

- MOET blastocyst: GSE12327 and GSE21030.
- IVP blastocyst: GSE24596 and GSE24936
- MOET and IVP blastocysts and day 13 conceptuses: GSE27817
- MOET and IVP day 16 conceptuses: GSE40101

Gathering all the samples, the final sample sizes per IVP or MOET groups were: blastocysts, n = 9; day 13 embryos, n = 3; and day 16 embryos, n = 3; per group.

All these studies employed the Affymetrix Bovine Genome Array platform for hybridization. The raw data obtained from all samples were processed with the gcRMA package [39]. Data were imported into R, background corrected, and then transformed and normalized using the quantile normalization method. Next, rows of each dataset were collapsed to retain the microarray probe with the highest mean value from the group of genes with the same official symbol. Batch effects (i.e., the fact that data were obtained from different studies) were removed with the ComBat function from the sva package [40].

#### Epigenomic data

Datasets related to MOET and IVP blastocysts were collected from four datasets: GSE128354, GSE101895, GSE97517 and GSE69173. Data from the TE of day 17 embryos were derived from a previous study [35]. These studies employed the two-channel 400 K EmbryoGENE DNA Methylation Array. Following the quality check of each sample [41], the sample size for the IVP and MOET groups was: blastocyst, n = 8; and day-17 conceptuses, n = 4; per group.

### Step 2. Determination of temporally differentially expressed genes (DEG) and differentially methylated regions (DMR)

Identification of DEG and DMR was performed with the limma R package [42]. To identify genes that behaved differently over time in the IVP embryos relative to the MOET embryos, contrasts were made between day 16 and day 13 conceptuses, and between day 13 conceptuses and blastocysts in both groups. For determination of DMR, a previously developed pipeline [41] was adapted to the present study. Briefly, the M-values and A-values were determined in the probes that were above the background level. These values were employed for a within-array loess normalization. Next, a between array quantile normalization was applied to ensure that the intensities had the same empirical distribution across arrays and across channels. The linear model was fit to the individual log-intensities for each probe using the *lmscFit* function. This last modification was done to analyse the channels separately, as described previously [43]. Next, contrasts were made between day 17 conceptuses and blastocysts in the IVP vs MOET groups. For the sake of comparisons, and since the subsequent analyses validated the findings, a p-value <0.05 and a fold change higher than 1 were employed as statistical criteria for both DEG and DMR.

### Step 3: Identification of common DEG and differentially methylated genes (DMG)

Genes in the corresponding exons of DMR were defined as DMG. The DMG that overlapped with the DEG were also methylated in the promoter and/or the intron (S1 Fig) and presumably, these regions were extensively methylated. If a gene showed more than one differentially methylated region, only the region with the lowest p-value was considered. DEG and DMG that were differentially expressed *and* differentially methylated over time in IVP relative to MOET embryos were identified by Venn Diagram and were subjected to a hierarchical clustering according to their expression profile in day 13 and day 16 conceptuses, using Spearman Rank Correlation as similarity metric and complete linkage as clustering method, implemented with the Cluster 3.0 software [44]. The resulting dendrogram and the heat map were visualized with Java TreeView [45].

Genes in each of the resulting clusters were evaluated through a functional analysis with the DAVID software [46]. The *Bos taurus* protein-coding genome was selected as background. The goal of this step was to determine the top enriched annotation terms in the Functional Annotation Chart (p<0.01). Additionally, the proportion of hyper- or hypo-methylated DMG was assessed in each cluster, and the probability of such a proportion, given the proportions in all the DMR, was estimated through a hypergeometric test. The relative methylation levels for specific genes in each group were estimated from the fitted coefficients generated from the application of the *lmscFit* function of the limma package [42].

Moreover, the GSE56513 dataset was employed to verify the consistency of the expression profiles for genes in the resulting clusters. This dataset corresponds to a study where mRNA was extracted from MOET embryos at days 7 and 10, and embryos after AI at days 13, 16 and 19; and measured using RNAseq [47]. Data in RPKM from embryos at days 7, 13 and 16 were downloaded and log2 transformed. Afterwards, the trajectories of average expression for genes in the resulting clusters were plotted.

### Predictions in independent datasets

With the aim of validating our results, the expressions of significant genes sets were employed to make predictions in independent datasets. For this, the following datasets were selected and downloaded from the GEO database:

- GSE75750: Corresponding to a study where the transcriptome of short and long age-matched day 15 conceptuses derived from MOET was compared [48]. Short conceptuses (n = 5) were ovoid in shape, with a length <10 mm. Long conceptuses (n = 5) were early tubular in morphology, with a length between 30 and 100 mm. This study employed the Affymetrix Bovine Genome Array platform for hybridization of the extracted embryonic mRNA. The raw data was processed as described for the transcriptomic data in Step 1.
- GSE126680: this dataset corresponds to a study in which IVP embryos were treated or not with 100 ng/ml of recombinant human Dickkopf-related protein 1 (DKK1) from day 5 to 7.5 of culture [49]. After transfer, conceptuses were recovered at day 15 of gestation and their lengths were measured. In the present study, only conceptuses with similar length were employed (DKK1, n = 5, length: 112 ± 33 mm; control, n = 5, length: 100 ± 26 mm). The extracted mRNA molecules were quantified through RNAseq. The raw counts were transformed through the variance stabilizing transformation method [50], using the *vst* function from the DESeq2 package [51] for R.

For the prediction, three models were constructed with the following groups of genes: temporally DEG, overlapped DEG/DMG (i.e., genes that were both differentially expressed and differentially methylated), and one of the gene clusters resulting from the clustering of the overlapped DEG/DMG. The training set consisted of the expression of those three groups of genes in the day 13 and day 16 conceptuses, while the expression in the short/long conceptuses and DKK1/control conceptuses constituted the testing sets. An add-on batch effect adjustment of the testing data with the training data was performed with the bapred package [52]. Support vector machine with linear kernels was used as a classifier, employing the leave-one-out cross validation (LOOCV) method as the internal control. The LOOCV was run with 100 resampling interactions. The unknown sample was predicted with 100% accuracy in every case, with a cost ranging from 0.1 to 2. The algorithm was applied with the kernlab package [53], through the caret package [54] for the R software. The accuracy of each prediction is reported.

## Results

### Temporal DEG and DMR from day 7 blastocyst to elongated conceptus in IVP- and MOET-derived embryos

The numbers of DEG and DMR were 1535 and 43928, respectively. For the DMR, 19562 genes were differentially hypermethylated and 24366 were differentially hypomethylated in the IVP group relative to the MOET group from the blastocyst stage to the day 17 conceptus. The absolute proportions of hypermethylated CpG islands and exonic regions were greater for the IVP group than the MOET group (S2 Fig). Accordingly, there were 6360 DMG (i.e, genes that were differentially methylated in their exonic regions), corresponding to 4495 (70.7%) hypermethylated regions in the IVP group and 1865 regions (29.3%) in the MOET group.

### Genes differentially changing both in expression *and* methylation levels from blastocyst to elongated conceptus in IVP- versus MOET-derived embryos

**Fig 2:**
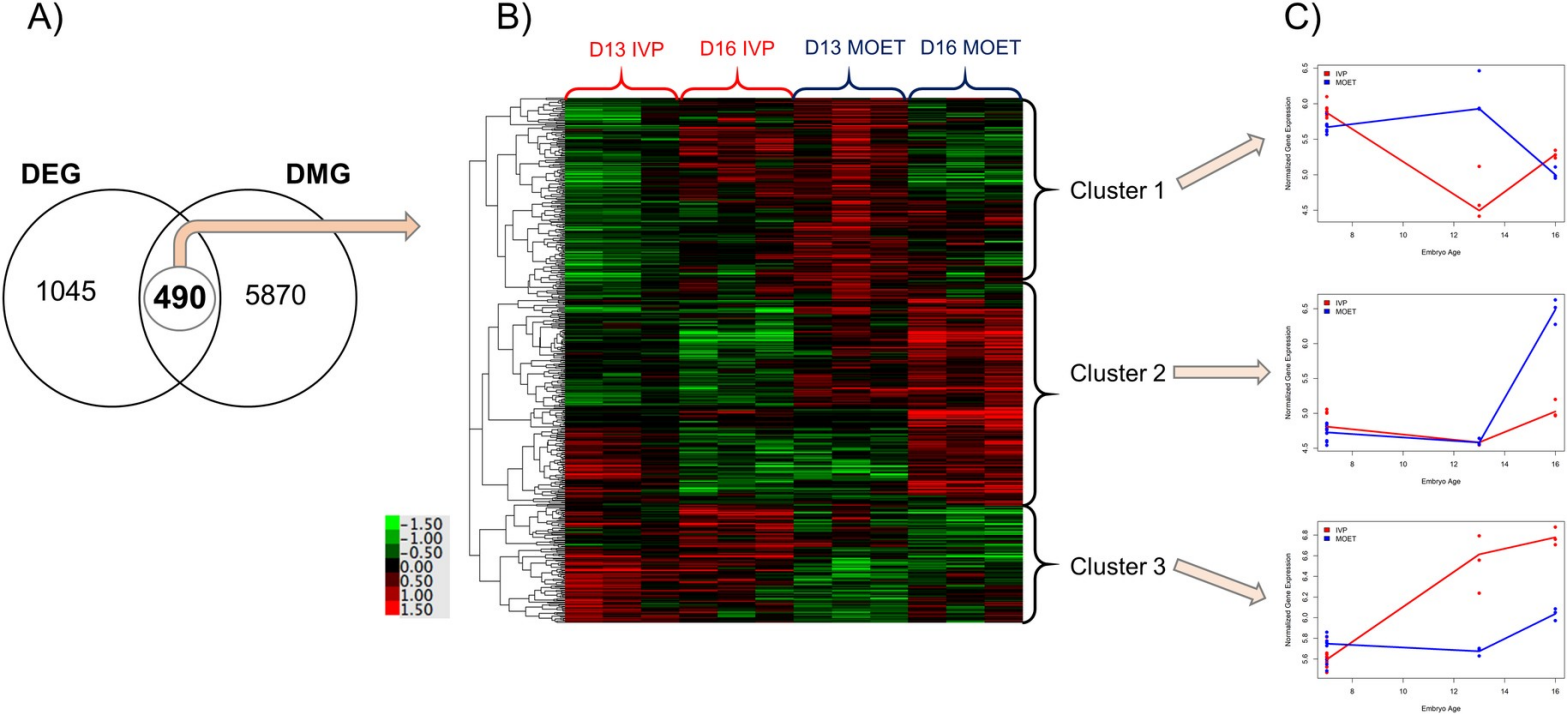
Identification of temporal genes both differentially expressed and differentially methylated between in *vitro* and in *vivo* produced embryos. A) Venn diagram showing the overlap between differentially expressed genes (DEG) and differentially methylated genes (DMG). DEG and DMG were defined as those genes or exonic regions changing differentially from blastocyst to elongation between *in vivo* and *in vitro* groups. B) Hierarchical clustering and heat map of the expressions of the 490 overlapped genes in day 13 (D13) and D16 embryos produced *in vitro* (IVP) or *in vivo* (after ovarian superovulation followed by embryo collection and transfer; MOET). C) Expression trajectories for genes in each of the main determined clusters, at 7, 13 and 16 days of embryo age, in IVP (red lines) or MOET (blue lines) groups.

**Fig 3:**
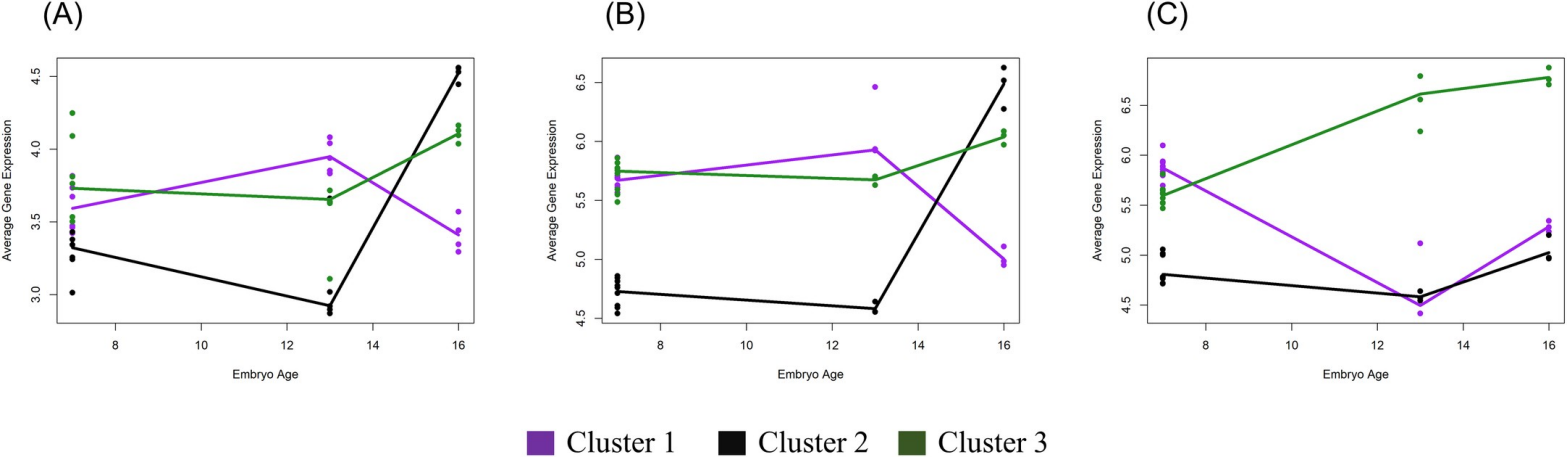
Trajectories of average expression for genes in the three clusters. Clusters were determined as illustrated in Fig 2. (A) Gene expression was obtained from an external and independent dataset from embryos obtained after artificial insemination, except for day-7 MOET embryos. (B) Gene expression in MOET embryos. (C). Gene expression in IVP embryos. MOET: embryos produced in vivo (ovarian superovulation followed by embryo collection and transfer). IVP: embryos produced in vitro. Cluster 1: purple lines. Cluster 2: black lines. Cluster 3: green lines.

The main results of this step are illustrated in Fig 2. The Venn Diagram (panel A) depicts 490 genes that were both differentially expressed and hyper -or hypo- methylated from the blastocyst stage to the elongated conceptus in IVP-derived compared to MOET-derived embryos. The heat map and dendrogram resulting from the hierarchical clustering of these 490 genes according to their expression in day 13 and day 16 conceptuses are shown in panel B. There were three main clusters that presented the following average expression patterns in the IVP conceptuses compared to the MOET conceptuses (panel C). Cluster 1: 188 genes decreasing in expression by day 13 but increasing again by day 16. Cluster 2: 191 genes with similar expression by day 13 but lower expression by day 16. Cluster 3: 111 genes with increased expression by day 13 and day 16. The list of genes in each cluster is detailed in S1 Table. Accordingly, the expression profile for genes in these clusters in MOET embryos followed similar trajectories to those in AI-derived embryos, underlining the consistency of the results (Fig 3). The top enriched annotation terms (p<0.01) in each of the three clusters are shown in Table 1. The KEGG pathway "focal adhesion" was the top enriched pathway in Cluster 1 (FDR = 0.003), followed by the cellular component: "extracellular exosome" (FDR = 0.04). This last term was the top enriched term in Cluster 2 (FDR<0.0001), followed by UniProtKB keyword "Phosphoprotein" (FDR = 0.01). Genes in Cluster 3 also enriched the "extracellular exosome" term, but this enrichment was not significant at the adjusted p-value (FDR = 0.6).

**Table 1:** Top enriched annotation terms (p<0.01) in each of the three clusters.

Category	Term	Genes	%	P-value	FDR
**Cluster 1**					
KEGG_PATHWAY	Focal adhesion	13	7	0.000019	0.0033
GOTERM_CC	extracellular exosome	43	23	0.000220	0.0410
UP_KEYWORDS	Actin-binding	8	4.3	0.000440	0.0870
GOTERM_BP	oxidation-reduction process	13	7	0.000510	0.4100
KEGG_PATHWAY	ECM-receptor interaction	7	3.7	0.001000	0.0900
UP_KEYWORDS	Cytoplasm	32	17.1	0.001300	0.1200
UP_KEYWORDS	Lipid biosynthesis	6	3.2	0.001900	0.1300
GOTERM_CC	basement membrane	5	2.7	0.002600	0.1900
GOTERM_CC	ruffle	5	2.7	0.004400	0.1900
GOTERM_CC	mitochondrial matrix	7	3.7	0.004900	0.1900
GOTERM_CC	extracellular space	21	11.2	0.005100	0.1900
GOTERM_MF	actin-dependent ATPase activity	3	1.6	0.005500	1.0000
GOTERM_CC	T cell receptor complex	3	1.6	0.006500	0.2000
UP_KEYWORDS	Oxidoreductase	11	5.9	0.007500	0.3500
UP_KEYWORDS	Myosin	4	2.1	0.009500	0.3500
GOTERM_BP	post-embryonic development	5	2.7	0.009600	1.0000
**Cluster 2**					
GOTERM_CC	extracellular exosome	49	26.1	0.0000005	0.0000920
UP_KEYWORDS	Phosphoprotein	47	25	0.0000780	0.0150000
UP_KEYWORDS	Glycoprotein	25	13.3	0.0006300	0.0610000
KEGG_PATHWAY	Lysosome	8	4.3	0.0008300	0.1700000
GOTERM_CC	early endosome	7	3.7	0.0041000	0.3900000
UP_KEYWORDS	Lysosome	6	3.2	0.0043000	0.2100000
GOTERM_CC	membrane raft	6	3.2	0.0068000	0.4300000
KEGG_PATHWAY	Glycolysis / Gluconeogenesis	5	2.7	0.0071000	0.5100000
UP_KEYWORDS	Disulfide bond	28	14.9	0.0080000	0.3100000
**Cluster 3**					
GOTERM_CC	extracellular exosome	25	22.3	0.0056	0.6900

The annotation terms were estimated with the Functional Annotation Chart of the DAVID software. GOTERM: Gene Ontology term. CC: cellular component. BP: biological process. MF: molecular function. UP_KEYWORDS: UniProtKB keywords.

**Fig 4:**
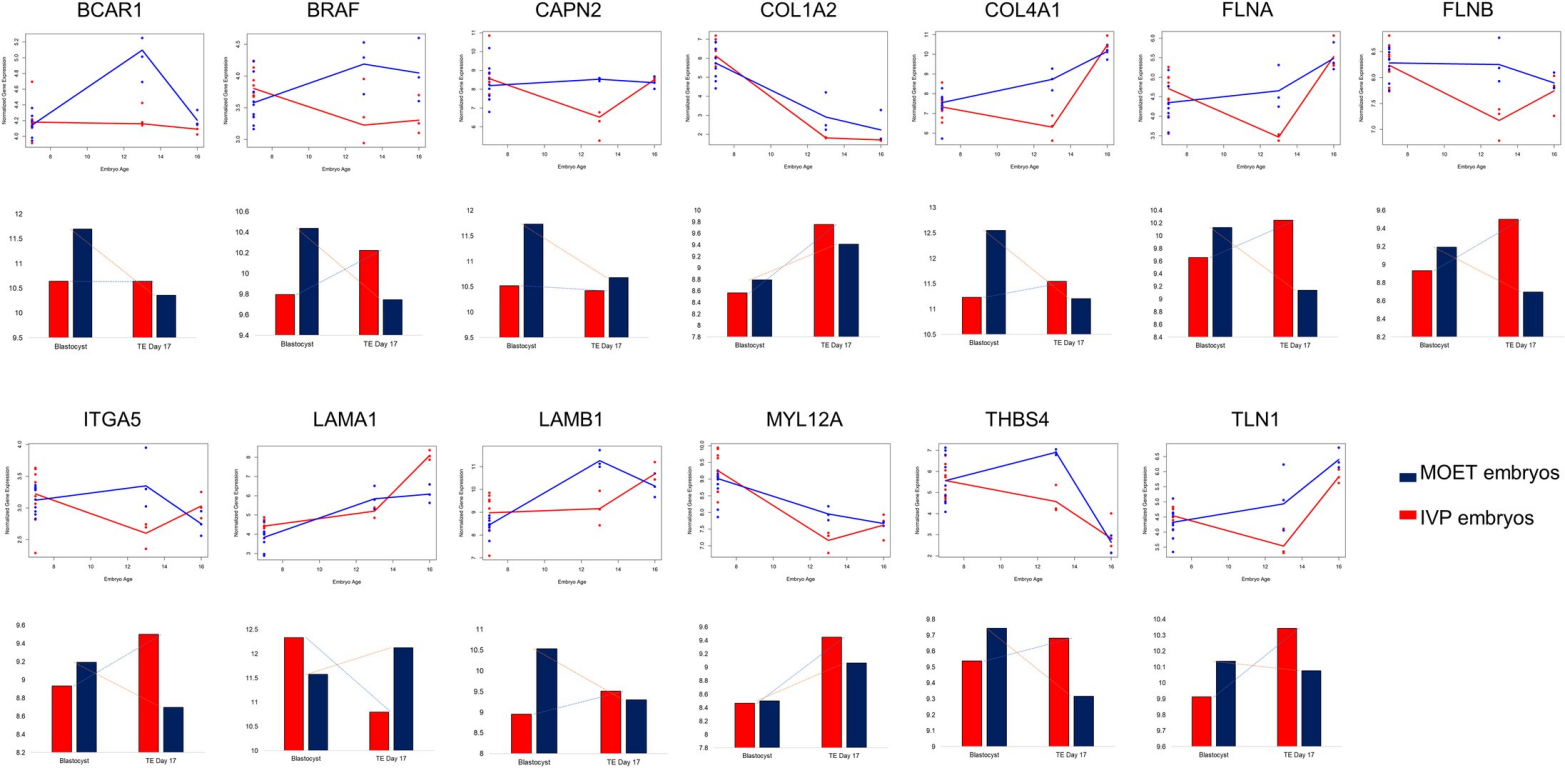
Differences in expression (line charts) and methylation (bar charts) levels in embryos produced *in vitro* or *in vivo*, from blastocyst to elongation. The expression levels are shown for blastocyst, day 13 and day 16 stages. The methylation levels are depicted for the blastocyst and the trophectoderm (TE) of day 17 embryos. Analysed genes are those involved in the "focal adhesion" KEGG pathway, which were part of the Cluster 1, determined as illustrated in Fig 2. *BCAR1*: Cas family scaffolding protein; *BRAF*: B-Raf proto-oncogene, serine/threonine kinase; *CAPN2*: calpain 2, (m/II) large subunit; *COL1A2*: collagen type I alpha 2 chain; *COL4A1*: collagen type IV alpha 1 chain; *FLNA*: filamin A; *FLNB*: filamin B; *ITGA5*: integrin subunit alpha 5; *LAMA1*: laminin subunit alpha 1; *LAMB1*: laminin subunit beta 1; *MYL12A*: myosin, light chain 12A, regulatory, non-sarcomeric; *THBS4*: thrombospondin 4; *TLN1*: talin 1. Blue lines or bars correspond to embryos produced *in vivo* (after ovarian superovulation; MOET). Red lines or bars represent embryos produced *in vitro* (IVP).

The number and proportion of genes hypermethylated in the IVP or MOET groups, respectively, in each cluster, were as follows–Cluster 1: 151 (80.3%) and 37 (19.7%); Cluster 2: 130 (68.1%) and 61 (31.9%); Cluster 3: 78 (70.3%) and 33 (29.7%). From the three clusters, only Cluster 1 presented a significantly higher proportion of hypermethylated genes in the IVP group, given the proportions of methylation in the exons of the DMR (p = 0.001). Furthermore, the differences in expression and methylation levels for each of the genes in Cluster 1 enriching the "focal adhesion" KEGG pathway from blastocyst to elongated conceptus are shown in Fig 4.

### Predictions in independent datasets

The overall rationale behind this analysis was the following: given that a delayed development would be manifested in reduced conceptus length, and that DKK1 treatment improves embryo survival, the expression pattern of at least some genes should resemble those of IVP conceptuses in the first case, and MOET conceptuses in the second. Thus, the hypotheses linked with these predictions, based on expression data of certain genes from day 15 embryos, were the following for each dataset. For GSE75750: short and long conceptuses will be predicted to be IVP and MOET embryos, respectively, while for GSE126680, DKK1-treated and non-treated embryos will be predicted to be MOET and IVP conceptus, respectively.

Next, we show the results of each prediction, and the corresponding accuracy according to the hypotheses, using the expression from the following genes to construct each model:

- 1535 DEG: the accuracy of the predictions was 70% for samples from both datasets, which was not significant (p = 0.17). Therefore, these genes do not have a comparable expression pattern between IVP and short MOET embryos or between MOET and DKK1-treated embryos.
- 490 overlapping DEG and DMG: for GSE75750 samples, all the short conceptuses and four out of five long conceptuses were predicted as IVP and MOET conceptuses, respectively, giving an accuracy of 90% (p = 0.01). For GSE126680, four out of five of the DKK1-treated and non-treated embryos were predicted as MOET and IVP embryos, respectively, with an accuracy of 80% (p = 0.054).
- Genes in Cluster 1: The accuracy of the prediction was 100% for samples from GSE75750, since all the short and long conceptuses were classified as being derived from IVP and MOET, respectively (p = 0.005). For samples from GSE126680, all the DKK1-treated embryos and four out of five control embryos were predicted as MOET and IVP conceptuses, respectively, with an accuracy of 90% (p = 0.01). Predictions with the other two clusters did not reach such a high accuracy (GSE75750: Cluster 2: 66%, p = 0.37; Cluster 3: 70%, p = 0.17. GSE126680: Cluster 2: 70%, p = 0.17; Cluster 3: 50%, p = 0.62).

## Discussion

Embryo transfer technology has been used in cattle more intensively than in any other livestock species [4]. The number of bovine embryos that are transferred worldwide is constantly increasing [55], and thus, for the past few years the number of IVP embryos transferred has surpassed the number produced by MOET. Indeed, data collated by the International Embryo Technology Society illustrate this divergent trend regarding the type of production: a decrease (-17.5%) in the number of *in vivo*-derived embryos whereas the production of IVP embryos reached a plateau (+0.2%), being 71% of the reported transferred embryos worldwide in 2019 [4]. Nevertheless, the lower performance of IVP embryos compared to MOET embryos to sustain pregnancy is critical for the industry and remains a hot topic of debate; despite much research, a complete understanding of the underlying issues involved in the suboptimal competency of IVP embryo is still lacking.

The current meta-analysis of available transcriptomic and epigenomic data from IVP and MOET embryos at different key stages of development will advance the field. However, this study presents challenges as embryos were produced in distinct environments, using, for example, different parental genetic combinations, culture conditions and superovulatory treatments. Furthermore, the combination of different datasets precludes the application of methods that requires the omics techniques to be measured in the same individual, and so, our approach consisted of single-omics analysis and posterior correlation. Nevertheless, and precisely because of the heterogeneity of the data, the results reported here can be interpreted as those occurring consistently because of the *in vitro* procedure, or more probably, the lack of oviductal environment during the first days, which impacts the subsequent embryonic development of the TE [56].

In the present study, results obtained from the re-analysis of the transcriptomic data were compared to the epigenomic data, to identify genes for which expression and DNA methylation patterns from blastocyst to elongated conceptus changed differentially between IVP and MOET embryos, which could impact the TE function. This embryonic region was chosen because differential DNA methylation was found to be prominent at intragenic sequences within the TE of IVP and MOET day 17 conceptuses, but not in the embryonic disc [35]. In addition, the smaller size observed in IVP embryos at day 13 [9], compared to MOET counterparts, may reflect a defect in elongation, potentially caused by errors in the TE.

Comparison of DEG and DMG resulted in the identification of 490 genes that were classified in three clusters according to their expression in day 13 and day 16 conceptuses (Fig 2A and 2B). To discern the variation more clearly in expression in the embryos undergoing elongation, samples from blastocysts were not considered for the hierarchical clustering. However, the plots of the expression levels for each cluster depict the pattern at the 3 time points, i. e., day 7, day 13 and day 16 of embryonic age (Fig 2C). The first cluster, Cluster 1, was constituted by genes that showed a remarkable difference in expression at day 13 between the IVP and MOET groups. Specifically, these genes, on average, decreased in expression in the IVP conceptuses at day 13, and increased again at day 16. In contrast, genes in Cluster 2 exhibited a similar expression at day 13 between groups but then strongly increased in expression in day 16 MOET-derived conceptuses. Lastly, genes in Cluster 3 were more highly expressed in IVP conceptuses than MOET conceptuses at both days 13 and 16. Notoriously, the comparable expression profiles of genes in the three clusters between MOET conceptuses and conceptuses produced after AI in an independent experiment (Fig 3), validate the pipeline followed to identify these genes and the reproducibility of the results reported here. In addition, they suggest that these genes could behave in a similar fashion in embryos produced *in vivo* but not *in vitro*, and they are weakly influenced by other factors such as the parental genetic combination. However, it is worth noting that the endometrium is sensitive to the embryo characteristics, since for example, both the origin [14] and length of the embryos [7] modify the endometrial transcriptome. Therefore, the maternal environment could influence the embryonic transcriptome as well.

The functional annotation analysis for genes in each of the identified clusters revealed that genes in Cluster 1 significantly enriched for the "focal adhesion" pathway (FDR<0.05). In addition, the cellular component "extracellular exosome" was enriched by genes in both Clusters 1 and 2 (FDR<0.05) and involved 23% and 26 of the genes in the Clusters 1 and 2, respectively. The expression pattern of genes encoding for adhesion proteins is critical to establish the biological shape and structure of the embryos since they maintain the polarity of cell associations with their neighbours and the surrounding extracellular matrix (ECM) [57]. Furthermore, remodelling of the actin cytoskeleton, a key component of eukaryotic cells, is achieved through actin-binding proteins [58], which was another term significantly enriched in Cluster 1 (FDR<0.1). Therefore, focal adhesion is essential for cytoskeletal organization, differentiation, proliferation, and survival of the embryo [59, 60]. A closer evaluation of the expression and methylation profiles of the 13 genes from Cluster 1 involved in the "focal adhesion" pathway (Fig 4) showed that for almost all the genes (except for *MYL12A*) the methylation pattern differed at the blastocyst stage. Interestingly, previous work reported that culture conditions of IVP embryos de-regulated the focal adhesion pathway at the blastocyst stage [61]. Furthermore, supplementation of the culture media with epidermal growth factor (EGF) and hyaluronic acid (HA) during or after embryonic genome activation altered the expression and DNA methylation patterns of genes involved in this pathway [62]. Stimulation of growth factors, such as EGF, and adhesion to the ECM is required for normal cell growth [63], and HA is one of the main components of ECM, that can improve the blastocyst rate of IVP bovine embryos [64]. Thus, the deregulation of genes involved in the focal adhesion pathway impacts embryonic developmental competence and quality.

In the present study, all genes from Cluster 1 involved in this pathway (except for *COL1A2*, *MYL12A* and *LAMA1*) showed a similar DNA methylation pattern: it increased or decreased from the blastocyst stage to the day 17 conceptus for IVP or MOET embryos, respectively. For *COL1A2* and *MYL12A*, DNA methylation pattern increased with embryo age in both groups, while for *LAMA1*, it decreased or increased from blastocyst to day 17 conceptus for IVP or MOET embryos, respectively. DNA methylation in gene bodies is surprisingly abundant and has been demonstrated to be positively correlated with gene expression [65]. However, gene expression levels are better inversely correlated with the methylation of the first exon than with that of the promoter [31]. Therefore, intragenic DNA methylation is involved in the regulation of gene expression, and its inhibition or suppression depends on the genomic region [66]. Here, the exact relationship between DNA methylation and gene expression cannot be directly estimated since changes in DNA methylation were calculated from the blastocyst to day 17 conceptus, while gene expression was assessed from blastocyst to day 13 and day 16 conceptus. Thus, it is not possible to dissect the dynamics of DNA methylation between the blastocyst and day 17 conceptus. One possibility is that the increasing methylation toward day 17 for genes in the focal adhesion pathway in IVP embryos was related with the "normalization" in the expression toward day 16, since, at day 13, the difference in expression between IVP and MOET embryos was remarkable but not at day 16. For *COL1A2* and *MYL12A*, the DNA methylation pattern seemed to be inversely correlated with gene expression; while for *LAMA1*, the hypomethylation at day 17 of IVP embryos compared to MOET embryos was probably related with a higher expression at day 16.

In addition to focal adhesion, the other term significantly enriched in both Cluster 1 and Cluster 2 (FDR<0.05), with around a quarter of the genes in those clusters, was "extracellular exosome". This term was enriched in Cluster 3 as well, but with an FDR = 0.6. For Cluster 1, seven out of the 13 genes involved in focal adhesion (*CAPN2*, *COL1A2*, *FLNA*, *FLNB*, *LAMB1*, *TLN1* and *THBS4*) were part of this cellular component. Exosomes are vesicles released into the extracellular region by fusion of the limiting endosomal membrane of a multivesicular body with the plasma membrane [67]. These vesicles can be secreted by oviductal and endometrial cells [68], and also by IVP and *in vivo* derived embryos [69, 70]. They can play essential roles in maternal-embryo communication [71]. Extracellular vesicles derived from trophoblast cells from the conceptus at day 15 and 17 have been shown to contain interferon-tau, in addition to bioactive molecules that modulate the adhesion pathway [70, 72]. Therefore, these vesicles participate in embryo–maternal interactions during early embryonic development and the maternal recognition of pregnancy, although the temporal expression of the gene encoding for interferon-tau did not differ between IVP and MOET embryos in this study (S3 Fig). Interestingly, genes that encoded for extracellular vesicles were related to (p<0.05): ECM receptor interaction, collagen, and epidermal growth factor in Cluster 1 (genes downregulated at day 13 in IVP embryos); glycoprotein and glycolysis/gluconeogenesis in Cluster 2 (genes more expressed at day 16 in MOET conceptuses) and protein acetylation in Cluster 3 (genes more expressed at day 13 and day 16 in IVP conceptuses).

The final step in this study was to underpin our results through the application of machine learning methods, to make predictions in data from day 15 IVP and MOET conceptuses under different conditions. The hypothesis tested with these predictions was that short (i.e., potentially developmentally compromised) MOET conceptuses and IVP embryos treated with DKK1 (i.e., developmentally superior) would be predicted as IVP and MOET embryos, respectively, according to the expression of at least some genes. The accuracy of the predictions improved when using the expression of the overlapping genes between DEG and DMG, compared to using only the DEG, highlighting the value of combining two "omics" technologies rather than a single one, to explore biological events. Furthermore, using the expression of the 188 genes in Cluster 1, in the day 13 and day 16 IVP and MOET conceptuses to train the model, the accuracies of the predictions were higher than 90% (p<0.05). That is, all short and long embryos were predicted as IVP and MOET, respectively, while all DKK1-treated embryos and four out five control embryos were predicted as MOET and IVP, respectively. This high accuracy could not be achieved with the genes in the other clusters, probably because the expression at day 16 has more relevance to stratify IVP and MOET embryos, and the models were tested with day 15 conceptuses.

These results are not suggesting that IVP embryos are always shorter or that DKK1-treated embryos would resemble MOET embryos. Rather, these findings support the notion that genes in Cluster 1, which show a strong deviation in their expression at day 13 between the groups, are important for conceptus elongation. In other words, IVP embryos could exhibit de-regulation of genes involved with development in the TE, while the early treatment with DKK1 would normalize the expression of those genes. In the study by Barnwell et al., [48], from which the data about short and long conceptuses were obtained, the authors found DEG in the long versus the short conceptuses were related to actin filaments of the cytoskeleton, consistent with genes in Cluster 1. Thus, inadequate elongation of IVP conceptuses could be a major contributing factor to early embryonic death seen during the peri-implantation period [73]. Predictions performed in the dataset obtained from the study of Tribulo et al. [49] indicate that treatment of IVP embryos with DKK1 between days 5 and 7.5, just before embryo transfer, could induce epigenomics modifications during the morula to blastocyst transition to regulate subsequent trophoblast elongation. Treatment with DKK1 significantly increased the length of filamentous conceptuses from 43.9 mm to 117.4 mm and the intrauterine content of interferon tau from 4.9 μg/ml to 16.6 μg/ml. In the present study, conceptuses of similar length derived from the transfer of embryos treated or not with DKK1 were selected. Therefore, the action of DKK1 probably involved the induction of the expression of genes related to focal adhesion as well, which in part can explain the increase in blastocyst development and pregnancy retention in DKK1-treated IVP embryos [74].

## Conclusion

This study applied a multi-omics data integration approach to identify genes that are both differentially expressed and methylated from the blastocyst to elongated conceptus stage, between IVP- and MOET-derived embryos. The integrated transcriptomic and epigenetics datasets were analysed using a set of bioinformatics methods and we evaluated the predictive ability of key genes using additional external data through machine learning methods. The results revealed a group of genes (Cluster 1) with a strong deviation in their expression between IVP and MOET embryos at day 13, when the elongation process is initiated. Several of these genes were significantly related to the focal adhesion pathway. Furthermore, their expression predicted less developed (short) day 15 MOET conceptuses as being IVP embryos and, conversely, day 15 IVP embryos treated with the progestomedin DKK1 as being MOET embryos. Therefore, the IVP process induces epigenomic and therefore transcriptomic changes in the early embryo that have consequences for subsequent conceptus elongation and focal adhesion, essential processes for survival. Results reported here can help in the understanding of some of the factors involved in the post-transfer pregnancy failures, that occur in cattle following the transfer of IVP embryos.

## Supporting information

S1 Fig Overlap of differentially expressed genes (DEG) and differentially methylated (DM) regions. The Venn diagram shows the overlap between DEG and DM exons, promoters and introns. The DM exons overlapping with the DEG were also methylated in the promoter and/or the intron (red oval), presumably corresponding to extensively methylated regions.
(TIF)

S2 Fig Main results from the epigenetic analysis. A) Volcano plot of showing the significant hypermethylated probes in each comparison (dark blue dots). B) and C) Absolute proportions of hypermethylated elements within significant probes, split by distance to the CpG islands (B) or gene region (C). The dotted line represents the baseline of this ratio when all selected probes are considered. TE: trophectoderm of day-17 embryos. BL: blastocyst. MOET: embryos produced in vivo (ovarian superovulation followed by embryo collection and transfer). IVP: embryos produced in vitro.
(TIF)

S3 Fig Temporal expression of the gene encoding for interferon-tau (IFN-tau). The plot shows similar expression trajectories for this gene at 7, 13 and 16 days of embryo age, in IVP (red lines) or MOET (blue lines) groups.
(TIF)

S1 Table List of genes in Clusters 1, 2, and 3. Clusters were determined as illustrated in Fig 2. Gene official symbol and gene names are shown, together with the fold change (FC) for the following contrasts: FC Diff D13: (day 13 IVP–day 7 IVP embryos)—(day 13 MOET–day 7 MOET embryos). FC Diff D16: (day 16 IVP–day 13 IVP embryos)—(day 16 MOET–day 13 MOET embryos). MOET: embryos produced in vivo (ovarian superovulation followed by embryo collection and transfer). IVP: embryos produced in vitro.
(XLSX)

## References

[1] Hansen PJ. Implications of Assisted Reproductive Technologies for Pregnancy Outcomes in Mammals. Annu Rev Anim Biosci. 2020;8: 395–413. 10.1146/annurev-animal-021419-084010 32069434

[2] Moore SG, Hasler JF. A 100-Year Review: Reproductive technologies in dairy science. J Dairy Sci. 2017;100: 10314–10331. 10.3168/jds.2017-13138 29153167

[3] Thibier M, Wagner HG. World statistics for artificial insemination in cattle. Livestock Production Science. 2002;74: 203–212.

[4] Viana J. 2019 Statistics of embryo production and transfer in domestic farm animals. Embryo Technology Newsletter. 2020;38: 7–26.

[5] Lonergan P, Fair T. In vitro-produced bovine embryos: dealing with the warts. Theriogenology. 2008;69: 17–22. 10.1016/j.theriogenology.2007.09.007 17950823

[6] Ealy AD, Wooldridge LK, McCoski SR. BOARD INVITED REVIEW: Post-transfer consequences of in vitro-produced embryos in cattle. J Anim Sci. 2019;97: 2555–2568. 10.1093/jas/skz116 30968113 PMC6541818

[7] Sánchez JM, Mathew DJ, Behura SK, Passaro C, Charpigny G, Butler ST et al. Bovine endometrium responds differentially to age-matched short and long conceptuses. Biol Reprod. 2019;101: 26–39. 10.1093/biolre/ioz060 30977805 PMC6614577

[8] Bazer FW, Thatcher WW. Chronicling the discovery of interferon tau. Reproduction. 2017;154: F11–F20. 10.1530/REP-17-0257 28747540 PMC5630494

[9] Lonergan P, Woods A, Fair T, Carter F, Rizos D, Ward F et al. Effect of embryo source and recipient progesterone environment on embryo development in cattle. Reprod Fertil Dev. 2007;19: 861–868. 10.1071/rd07089 17897589

[10] Barnwell CV, Farin PW, Whisnant CS, Alexander JE, Farin CE. Maternal serum progesterone concentration and early conceptus development of bovine embryos produced in vivo or in vitro. Domest Anim Endocrinol. 2015;52: 75–81. 10.1016/j.domaniend.2015.03.004 25917140

[11] Bertolini M, Beam SW, Shim H, Bertolini LR, Moyer AL, Famula TR et al. Growth, development, and gene expression by in vivo- and in vitro-produced day 7 and 16 bovine embryos. Mol Reprod Dev. 2002;63: 318–328. 10.1002/mrd.90015 12237947

[12] Mansouri-Attia N, Sandra O, Aubert J, Degrelle S, Everts RE, Giraud-Delville C et al. Endometrium as an early sensor of in vitro embryo manipulation technologies. Proc Natl Acad Sci U S A. 2009;106: 5687–5692. 10.1073/pnas.0812722106 19297625 PMC2667091

[13] Bauersachs S, Ulbrich SE, Zakhartchenko V, Minten M, Reichenbach M, Reichenbach HD et al. The endometrium responds differently to cloned versus fertilized embryos. Proc Natl Acad Sci U S A. 2009;106: 5681–5686. 10.1073/pnas.0811841106 19307558 PMC2666995

[14] Mathew DJ, Sánchez JM, Passaro C, Charpigny G, Behura SK, Spencer TE et al. Interferon tau-dependent and independent effects of the bovine conceptus on the endometrial transcriptome. Biol Reprod. 2019;100: 365–380. 10.1093/biolre/ioy199 30203055 PMC6378860

[15] Driver AM, Peñagaricano F, Huang W, Ahmad KR, Hackbart KS, Wiltbank MC et al. RNA-Seq analysis uncovers transcriptomic variations between morphologically similar in vivo- and in vitro-derived bovine blastocysts. BMC Genomics. 2012;13: 118. 10.1186/1471-2164-13-118 22452724 PMC3368723

[16] Corcoran D, Fair T, Park S, Rizos D, Patel OV, Smith GW et al. Suppressed expression of genes involved in transcription and translation in in vitro compared with in vivo cultured bovine embryos. Reproduction. 2006;131: 651–660. 10.1530/rep.1.01015 16595716

[17] Gad A, Besenfelder U, Rings F, Ghanem N, Salilew-Wondim D, Hossain MM et al. Effect of reproductive tract environment following controlled ovarian hyperstimulation treatment on embryo development and global transcriptome profile of blastocysts: implications for animal breeding and human assisted reproduction. Hum Reprod. 2011;26: 1693–1707. 10.1093/humrep/der110 21531990

[18] Heras S, De Coninck DI, Van Poucke M, Goossens K, Bogado Pascottini O, Van Nieuwerburgh F et al. Suboptimal culture conditions induce more deviations in gene expression in male than female bovine blastocysts. BMC Genomics. 2016;17: 72. 10.1186/s12864-016-2393-z 26801242 PMC4724126

[19] Kues WA, Sudheer S, Herrmann D, Carnwath JW, Havlicek V, Besenfelder U et al. Genome-wide expression profiling reveals distinct clusters of transcriptional regulation during bovine preimplantation development in vivo. Proc Natl Acad Sci U S A. 2008;105: 19768–19773. 10.1073/pnas.0805616105 19064908 PMC2604955

[20] Betsha S, Hoelker M, Salilew-Wondim D, Held E, Rings F, Grosse-Brinkhause C et al. Transcriptome profile of bovine elongated conceptus obtained from SCNT and IVP pregnancies. Mol Reprod Dev. 2013;80: 315–333. 10.1002/mrd.22165 23426952

[21] Clemente M, de La Fuente J, Fair T, Al Naib A, Gutierrez-Adan A, Roche JF et al. Progesterone and conceptus elongation in cattle: a direct effect on the embryo or an indirect effect via the endometrium? Reproduction. 2009;138: 507–517. 10.1530/REP-09-0152 19556439

[22] Zolini AM, Block J, Rabaglino MB, Rincon G, Hoelker M, Bromfield JJ et al. Genes associated with survival of female bovine blastocysts produced in vivo. Cell Tissue Res. 2020;382: 665–678. 10.1007/s00441-020-03257-y 32710275 PMC7686078

[23] Zolini AM, Block J, Rabaglino MB, Tríbulo P, Hoelker M, Rincon G et al. Molecular fingerprint of female bovine embryos produced in vitro with high competence to establish and maintain pregnancy. Biol Reprod. 2020;102: 292–305. 10.1093/biolre/ioz190 31616926 PMC7331872

[24] Urrego R, Rodriguez-Osorio N, Niemann H. Epigenetic disorders and altered gene expression after use of Assisted Reproductive Technologies in domestic cattle. Epigenetics. 2014;9: 803–815. 10.4161/epi.28711 24709985 PMC4065177

[25] Wu C, Morris JR. Genes, genetics, and epigenetics: a correspondence.[letter]. Science 2001;293(5532):1103–1105. 10.1126/science.293.5532.1103 11498582

[26] Li E, Zhang Y. DNA methylation in mammals. Cold Spring Harb Perspect Biol. 2014;6: a019133. 10.1101/cshperspect.a019133 24789823 PMC3996472

[27] Bird A, Taggart M, Frommer M, Miller OJ, Macleod D. A fraction of the mouse genome that is derived from islands of nonmethylated, CpG-rich DNA. Cell. 1985;40: 91–99. 10.1016/0092-8674(85)90312-5 2981636

[28] Ehrlich M, Lacey M. DNA methylation and differentiation: silencing, upregulation and modulation of gene expression. Epigenomics. 2013;5: 553–568. 10.2217/epi.13.43 24059801 PMC3864898

[29] Jones PA. Functions of DNA methylation: islands, start sites, gene bodies and beyond. Nat Rev Genet. 2012;13: 484–492. 10.1038/nrg3230 22641018

[30] Ma L, Muhammad T, Wang H, Du G, Sakhawat A, Wei Y et al. Putative promoters within gene bodies control exon expression via TET1-mediated H3K36 methylation. J Cell Physiol. 2020;235: 6711–6724. 10.1002/jcp.29566 31994732

[31] Brenet F, Moh M, Funk P, Feierstein E, Viale AJ, Socci ND et al. DNA methylation of the first exon is tightly linked to transcriptional silencing. PLoS One. 2011;6: e14524. 10.1371/journal.pone.0014524 21267076 PMC3022582

[32] Fair T. DNA methylation dynamics during oocyte and embryo development and its association with environmental induced alterations. Animal Reproduction. 2018; 13: 250–256.

[33] Salilew-Wondim D, Fournier E, Hoelker M, Saeed-Zidane M, Tholen E, Looft C et al. Genome-Wide DNA Methylation Patterns of Bovine Blastocysts Developed In Vivo from Embryos Completed Different Stages of Development In Vitro. PLoS One. 2015;10: e0140467. 10.1371/journal.pone.0140467 26536655 PMC4633222

[34] Wu C, Blondin P, Vigneault C, Labrecque R, Sirard MA. The age of the bull influences the transcriptome and epigenome of blastocysts produced by IVF. Theriogenology. 2020;144: 122–131. 10.1016/j.theriogenology.2019.12.020 31951983

[35] O’Doherty AM, McGettigan P, Irwin RE, Magee DA, Gagne D, Fournier E et al. Intragenic sequences in the trophectoderm harbour the greatest proportion of methylation errors in day 17 bovine conceptuses generated using assisted reproductive technologies. BMC Genomics. 2018;19: 438. 10.1186/s12864-018-4818-3 29866048 PMC5987443

[36] Barrett T, Wilhite SE, Ledoux P, Evangelista C, Kim IF, Tomashevsky M et al. NCBI GEO: archive for functional genomics data sets—update. Nucleic Acids Res. 2013;41: D991–5. 10.1093/nar/gks1193 23193258 PMC3531084

[37] Edgar R, Domrachev M, Lash AE. Gene Expression Omnibus: NCBI gene expression and hybridization array data repository. Nucleic Acids Res. 2002;30: 207–210. 10.1093/nar/30.1.207 11752295 PMC99122

[38] R Core Team. R: A Language and Environment for Statistical Computing. Vienna, Austria. 2020. http://www.r-project.org/index.html.

[39] Wu J, Irizarry R. gcrma: Background Adjustment Using Sequence Information. R package version 2.52.0. 2017

[40] Johnson WE, Li C, Rabinovic A. Adjusting batch effects in microarray expression data using empirical Bayes methods. Biostatistics. 2007;8: 118–127. 10.1093/biostatistics/kxj037 16632515

[41] Shojaei Saadi HA, O’Doherty AM, Gagné D, Fournier É, Grant JR, Sirard MA et al. An integrated platform for bovine DNA methylome analysis suitable for small samples. BMC Genomics. 2014;15: 451. 10.1186/1471-2164-15-451 24912542 PMC4092217

[42] Ritchie ME, Phipson B, Wu D, Hu Y, Law CW, Shi W et al. limma powers differential expression analyses for RNA-sequencing and microarray studies. Nucleic Acids Res. 2015;43: e47. 10.1093/nar/gkv007 25605792 PMC4402510

[43] Smyth GK, Altman NS. Separate-channel analysis of two-channel microarrays: recovering inter-spot information. BMC Bioinformatics. 2013;14: 165. 10.1186/1471-2105-14-165 23705896 PMC3673852

[44] de Hoon MJ, Imoto S, Nolan J, Miyano S. Open source clustering software. Bioinformatics. 2004;20: 1453–1454. 10.1093/bioinformatics/bth078 14871861

[45] Saldanha AJ. Java Treeview—extensible visualization of microarray data. Bioinformatics. 2004;20: 3246–3248. 10.1093/bioinformatics/bth349 15180930

[46] Huang da W, Sherman BT, Lempicki RA. Systematic and integrative analysis of large gene lists using DAVID bioinformatics resources. Nat Protoc. 2009;4: 44–57. 10.1038/nprot.2008.211 19131956

[47] Mamo S, Mehta JP, McGettigan P, Fair T, Spencer TE, Bazer FW et al. RNA sequencing reveals novel gene clusters in bovine conceptuses associated with maternal recognition of pregnancy and implantation. Biol Reprod. 2011;85: 1143–1151. 10.1095/biolreprod.111.092643 21795669

[48] Barnwell CV, Farin PW, Ashwell CM, Farmer WT, Galphin SP, Farin CE. Differences in mRNA populations of short and long bovine conceptuses on Day 15 of gestation. Mol Reprod Dev. 2016;83: 424–441. 10.1002/mrd.22640 27013032

[49] Tríbulo P, Rabaglino MB, Bo MB, Carvalheira LR, Bishop JV, Hansen TR et al. Dickkopf-related protein 1 is a progestomedin acting on the bovine embryo during the morula-to-blastocyst transition to program trophoblast elongation. Sci Rep. 2019;9: 11816. 10.1038/s41598-019-48374-z 31413296 PMC6694114

[50] Huber W, von Heydebreck A, Sueltmann H, Poustka A, Vingron M. Parameter estimation for the calibration and variance stabilization of microarray data. Stat Appl Genet Mol Biol. 2003;2: Article3. 10.2202/1544-6115.1008 16646781

[51] Love MI, Huber W, Anders S. Moderated estimation of fold change and dispersion for RNA-seq data with DESeq2. Genome Biol. 2014;15: 550. 10.1186/s13059-014-0550-8 25516281 PMC4302049

[52] Hornung R, Causeur D, Bernau C, Boulesteix AL. Improving cross-study prediction through addon batch effect adjustment or addon normalization. Bioinformatics. 2017;33: 397–404. 10.1093/bioinformatics/btw650 27797760

[53] Karatzoglou A, Smola A HK, Zeileis A. kernlab–An S4 Package for Kernel Methods in R. Journal of Statistical Software. 2004;11: 1–20.

[54] Kuhn M. Building predictive models in R using the caret package. Journal of Statistical Software. 2008;28: 1–26. 10.18637/jss.v028.i07 27774042 PMC5074077

[55] Hansen PJ, Block J. Towards an embryocentric world: the current and potential uses of embryo technologies in dairy production. Reprod Fertil Dev. 2004;16: 1–14. 10.10371/RD03073 14972098

[56] Rizos D, Ward F, Duffy P, Boland MP, Lonergan P. Consequences of bovine oocyte maturation, fertilization or early embryo development in vitro versus in vivo: implications for blastocyst yield and blastocyst quality. Mol Reprod Dev. 2002;61: 234–248. 10.1002/mrd.1153 11803560

[57] Shawky JH, Davidson LA. Tissue mechanics and adhesion during embryo development. Dev Biol. 2015;401: 152–164. 10.1016/j.ydbio.2014.12.005 25512299 PMC4402132

[58] Merino F, Pospich S, Raunser S. Towards a structural understanding of the remodeling of the actin cytoskeleton. Semin Cell Dev Biol. 2020;102: 51–64. 10.1016/j.semcdb.2019.11.018 31836290 PMC7221352

[59] Kaneko Y, Lecce L, Day ML, Murphy CR. Focal adhesion kinase localizes to sites of cell-to-cell contact in vivo and increases apically in rat uterine luminal epithelium and the blastocyst at the time of implantation. J Morphol. 2012;273: 639–650. 10.1002/jmor.20010 22322452

[60] Wrighton KH. Cell adhesion: the ‘ins’ and ‘outs’ of integrin signalling. Nat Rev Mol Cell Biol. 2013;14: 752. 10.1038/nrm3708 24263354

[61] Salilew-Wondim D, Saeed-Zidane M, Hoelker M, Gebremedhn S, Poirier M, Pandey HO et al. Genome-wide DNA methylation patterns of bovine blastocysts derived from in vivo embryos subjected to in vitro culture before, during or after embryonic genome activation. BMC Genomics. 2018;19: 424. 10.1186/s12864-018-4826-3 29859035 PMC5984773

[62] Saeed-Zidane M, Tesfaye D, Mohammed Shaker Y, Tholen E, Neuhoff C, Rings F et al. Hyaluronic acid and epidermal growth factor improved the bovine embryo quality by regulating the DNA methylation and expression patterns of the focal adhesion pathway. PLoS One. 2019;14: e0223753. 10.1371/journal.pone.0223753 31661494 PMC6818761

[63] Renshaw MW, Price LS, Schwartz MA. Focal adhesion kinase mediates the integrin signaling requirement for growth factor activation of MAP kinase. J Cell Biol. 1999;147: 611–618. 10.1083/jcb.147.3.611 10545504 PMC2151196

[64] Block J, Bonilla L, Hansen PJ. Effect of addition of hyaluronan to embryo culture medium on survival of bovine embryos in vitro following vitrification and establishment of pregnancy after transfer to recipients. Theriogenology. 2009;71: 1063–1071. 10.1016/j.theriogenology.2008.11.007 19157530

[65] Ball MP, Li JB, Gao Y, Lee JH, LeProust EM, Park IH et al. Targeted and genome-scale strategies reveal gene-body methylation signatures in human cells. Nat Biotechnol. 2009;27: 361–368. 10.1038/nbt.1533 19329998 PMC3566772

[66] Anastasiadi D, Esteve-Codina A, Piferrer F. Consistent inverse correlation between DNA methylation of the first intron and gene expression across tissues and species. Epigenetics Chromatin. 2018;11: 37. 10.1186/s13072-018-0205-1 29958539 PMC6025724

[67] Witwer KW, Buzás EI, Bemis LT, Bora A, Lässer C, Lötvall J et al. Standardization of sample collection, isolation and analysis methods in extracellular vesicle research. J Extracell Vesicles. 2013;2: 1. 10.3402/jev.v2i0.20360 24009894 PMC3760646

[68] Ng YH, Rome S, Jalabert A, Forterre A, Singh H, Hincks CL et al. Endometrial exosomes/microvesicles in the uterine microenvironment: a new paradigm for embryo-endometrial cross talk at implantation. PLoS One. 2013;8: e58502. 10.1371/journal.pone.0058502 23516492 PMC3596344

[69] Mellisho EA, Velásquez AE, Nuñez MJ, Cabezas JG, Cueto JA, Fader C et al. Identification and characteristics of extracellular vesicles from bovine blastocysts produced in vitro. PLoS One. 2017;12: e0178306. 10.1371/journal.pone.0178306 28542562 PMC5444795

[70] Nakamura K, Kusama K, Bai R, Sakurai T, Isuzugawa K, Godkin JD et al. Induction of IFNT-Stimulated Genes by Conceptus-Derived Exosomes during the Attachment Period. PLoS One. 2016;11: e0158278. 10.1371/journal.pone.0158278 27351483 PMC4924817

[71] Bridi A, Perecin F, Silveira JCD. Extracellular Vesicles Mediated Early Embryo-Maternal Interactions. Int J Mol Sci. 2020;21: 1163. 10.3390/ijms21031163 32050564 PMC7037557

[72] Kusama K, Nakamura K, Bai R, Nagaoka K, Sakurai T, Imakawa K. Intrauterine exosomes are required for bovine conceptus implantation. Biochem Biophys Res Commun. 2018;495: 1370–1375. 10.1016/j.bbrc.2017.11.176 29196267

[73] Wiltbank MC, Baez GM, Garcia-Guerra A, Toledo MZ, Monteiro PL, Melo LF et al. Pivotal periods for pregnancy loss during the first trimester of gestation in lactating dairy cows. Theriogenology. 2016;86: 239–253. 10.1016/j.theriogenology.2016.04.037 27238438

[74] Denicol AC, Block J, Kelley DE, Pohler KG, Dobbs KB, Mortensen CJ et al. The WNT signaling antagonist Dickkopf-1 directs lineage commitment and promotes survival of the preimplantation embryo. FASEB J. 2014;28: 3975–3986. 10.1096/fj.14-253112 24858280 PMC5395727

