# Supplementary figures and images for: Application of multi-omics data integration and machine learning approaches to identify epigenetic and transcriptomic differences between in vitro and in vivo produced bovine embryos
Source: PLoS One. 2021 May 24;16(5):e0252096. doi: 10.1371/journal.pone.0252096 (PMC8143403; doi:10.1371/journal.pone.0252096)

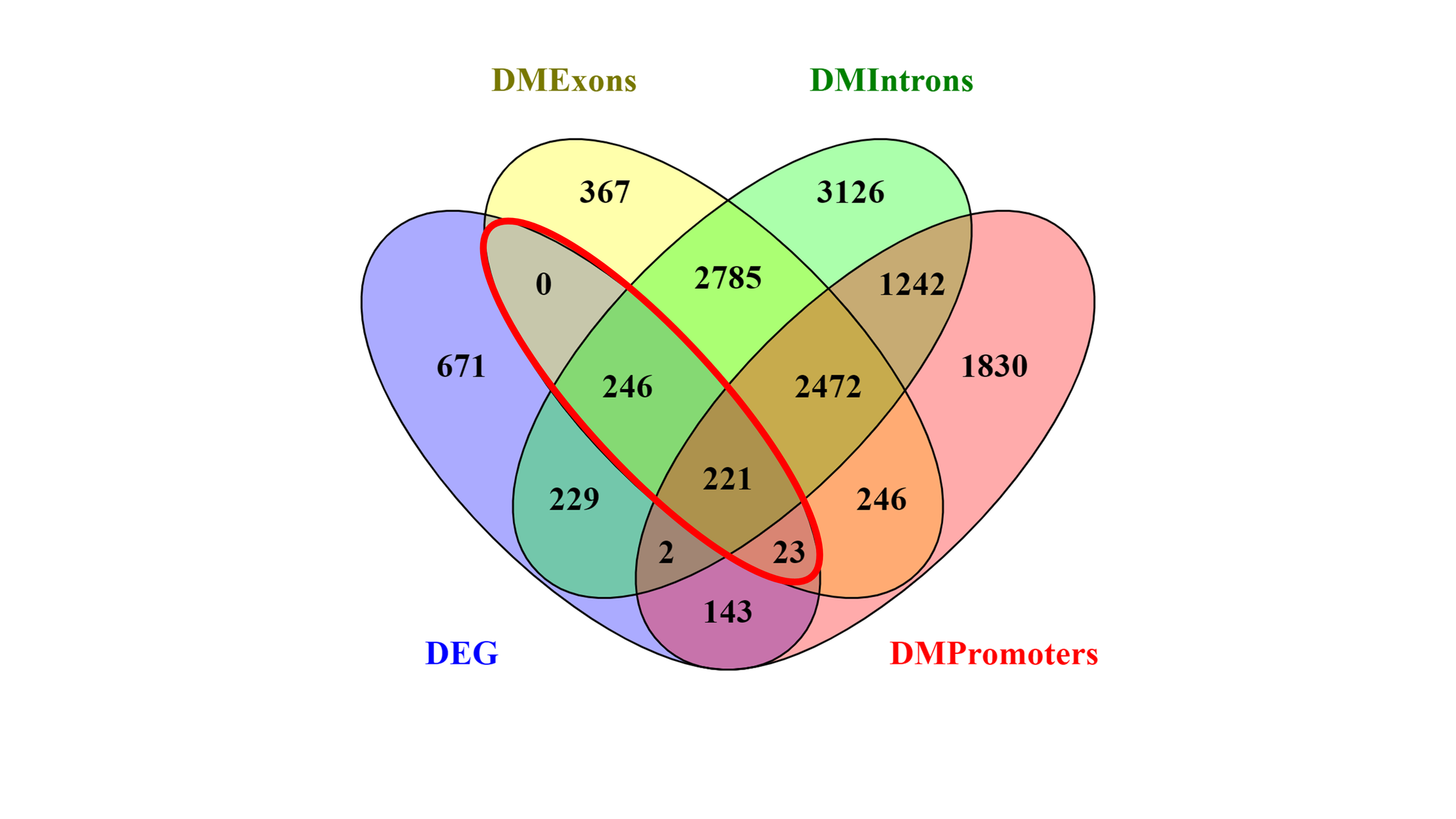

Supplement: S1 Fig — The Venn diagram shows the overlap between DEG and DM exons, promoters and introns. The DM exons overlapping with the DEG were also methylated in the promoter and/or the intron (red oval), presumably corresponding to extensively methylated regions. (TIF) [file pone.0252096.s001.tif]

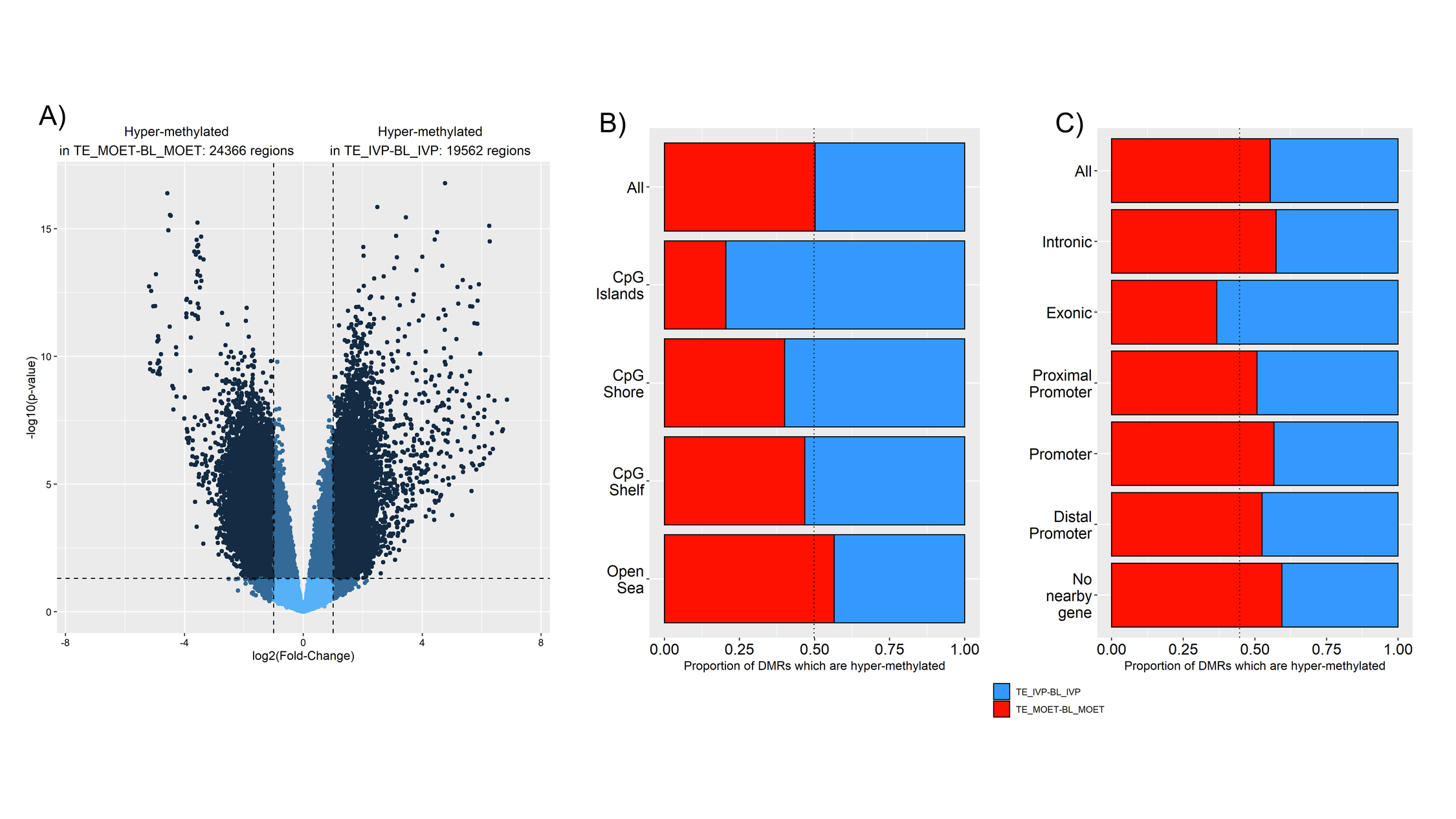

Supplement: S2 Fig — A) Volcano plot of showing the significant hypermethylated probes in each comparison (dark blue dots). B) and C) Absolute proportions of hypermethylated elements within significant probes, split by distance to the CpG islands (B) or gene region (C). The dotted line represents the baseline of this ratio when all selected probes are considered. TE: trophectoderm of day-17 embryos. BL: blastocyst. MOET: embryos produced in vivo (ovarian superovulation followed by embryo collection and transfer). IVP: embryos produced in vitro. (TIF) [file pone.0252096.s002.tif]

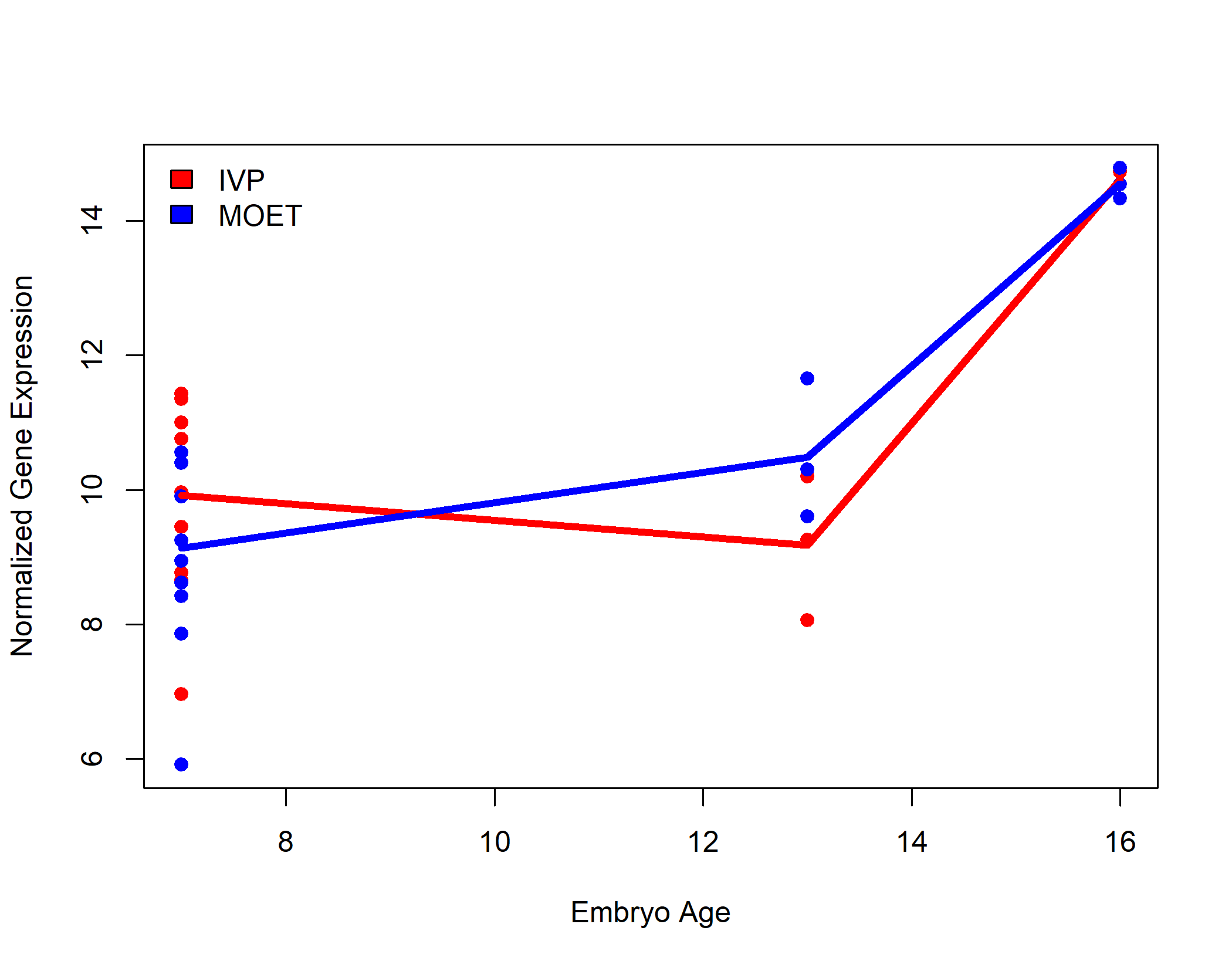

Supplement: S3 Fig — The plot shows similar expression trajectories for this gene at 7, 13 and 16 days of embryo age, in IVP (red lines) or MOET (blue lines) groups. (TIF) [file pone.0252096.s003.tif]
